# Machine learning-based infant crying interpretation

**DOI:** 10.3389/frai.2024.1337356

**Published:** 2024-02-08

**Authors:** Mohammed Hammoud, Melaku N. Getahun, Anna Baldycheva, Andrey Somov

**Affiliations:** ^1^Digital Engineering CREI, Skolkovo Institute of Science and Technology, Moscow, Russia; ^2^Engineering Department, University of Exeter, Exeter, United Kingdom

**Keywords:** time-series classification, Mel-frequency cepstral coefficient, spectrogram, machine learning, audio processing, time-series imagining

## Abstract

Crying is an inevitable character trait that occurs throughout the growth of infants, under conditions where the caregiver may have difficulty interpreting the underlying cause of the cry. Crying can be treated as an audio signal that carries a message about the infant's state, such as discomfort, hunger, and sickness. The primary infant caregiver requires traditional ways of understanding these feelings. Failing to understand them correctly can cause severe problems. Several methods attempt to solve this problem; however, proper audio feature representation and classifiers are necessary for better results. This study uses time-, frequency-, and time-frequency-domain feature representations to gain in-depth information from the data. The time-domain features include zero-crossing rate (ZCR) and root mean square (RMS), the frequency-domain feature includes the Mel-spectrogram, and the time-frequency-domain feature includes Mel-frequency cepstral coefficients (MFCCs). Moreover, time-series imaging algorithms are applied to transform 20 MFCC features into images using different algorithms: Gramian angular difference fields, Gramian angular summation fields, Markov transition fields, recurrence plots, and RGB GAF. Then, these features are provided to different machine learning classifiers, such as decision tree, random forest, K nearest neighbors, and bagging. The use of MFCCs, ZCR, and RMS as features achieved high performance, outperforming state of the art (SOTA). Optimal parameters are found via the grid search method using 10-fold cross-validation. Our MFCC-based random forest (RF) classifier approach achieved an accuracy of 96.39%, outperforming SOTA, the scalogram-based shuffleNet classifier, which had an accuracy of 95.17%.

## 1 Introduction

The motivation behind infant crying interpretation is to alleviate consequences that arise from continuous crying, such as discomfort, hunger, and sickness. Consequences such as disturbing crying, painful crying, and crying from sickness can also lead to serious health issues. The best solution that can relieve caregivers or inexperienced parents from this painful experience is being able to interpret the infant's crying. Additionally, it is considered helpful in detecting diseases such as hyperacusis, deafness, asphyxia, hypothyroidism, hyperbilirubinemia, and cleft palate.

Understanding of infant crying and the detection of the underlying facts is a wide research area in the signal processing and machine learning (ML) fields. Any infant cry signal always needs a translator to determine the reasoning, and the primary solution has been an interpretation made by someone who is always close to the infant. Although the potential of humans to understand infant cries should not be underestimated, a well-built computer system is better in some activities such as image (Ho-Phuoc, 2018) and audio classification (Zieliński et al., 2020).

According to Mukhopadhyay et al. (2013), the highest probability that people will understand an infant cry signal is 33.09%, whereas ML-based solutions can achieve 80.56% recognition accuracy. In cry audio signals, some features are not always present in healthy infants; however, they appear in infants with disease and discomfort (Farsaie Alaie and Tadj, 2012). Differentiating between cries is very challenging; for instance, the difference between a sleepy cry and a sick cry can be difficult for caregivers. Because of these reasons, a system that can solve this problem efficiently needs to be developed (Zayed et al., 2023).

The contribution of this study is as follows:

- Introduction of feature extraction using MFCC and several time-series imaging (TSI) methods, which have a higher feature representation capability for this type of signal.
- Many ML classifier methods are trained on these extracted features, and a comparison analysis is presented.

The novelty of this study is that the proposed method successfully classifies five classes of infant crying, including hunger, discomfort, belly pain, burping, and tiredness. The best results are achieved with the MFCC-based ML model, with an accuracy of 96.39% with testing data, outperforming state of the art.

## 2 Related work

Several studies have been proposed to prevent misunderstanding about infant crying, stress, and any serious medical problems. According to Liu et al. (2019), cry audio waveform and time-frequency analysis are conducted to extract features such as MFCC and Bark frequency cepstral coefficients (BFCCs). The final step of recognizing the infant cry is formulating features with a vertical vector and obtaining sparse representation using compressive sensing.

Deep learning (DL) and classical ML methods were explored (Cohen et al., 2020). An image of the infant's cry was obtained with Mel-scale representation as input to the convolutional neural network (CNN)-based model. A bidirectional recurrent neural network (RNN) was used to account for any temporal relationships between the input and output sequences of the audio data. For the classical ML, certain types of features were extracted, such as pitch-related, filter bank, and cepstrum coefficients, and logistic regression (LR) and support vector machine (SVM) were used as classifiers of infant crying.

DL-based methods are used in biomedical fields to help physicians understand the state of an infant. Lahmiri et al. (2022) built a system to classify infants into two classes: healthy and unhealthy. Cepstral features are extracted by the inverse discrete Fourier transform (DFT) of the log of DFT magnitude. These features are trained with CNN, deep feed-forward neural networks (DFFNN), and long short-term memory (LSTM).

In another study, a dataset of the expiration and inspiration of infant cry audio signals was collected (Matikolaie and Tadj, 2022). A system was built to identify whether the infant was sick or healthy. Next, features were extracted, including tilt, rhythm, intensity, and MFCCs. Some criteria were imposed, like considering the linguistic group of the infant's parent as the unborn infant learns those prosodic features in the last 3 months of pregnancy. Finally, the performance was evaluated by classification methods, such as SVM and decision tree (DT).

Le et al. (2019) used spectrogram images of the infant cry audio and trained them with several methods to detect abnormalities that arise in the first few months of an infant's life and could cause a permanent critical problem if not treated at an early stage. The methods used in this study were transfer learning, SVM, and ensemble learning. The ensemble combining transfer learning and SVM was more accurate than the individual ones.

Yao et al. (2022) proved that for a model to have high generalization, it needs to be trained in diverse data. This was cross-checked by building data with a controlled and uncontrolled environment, and the former data resulted in higher recall but high false positivity when testing was performed with data collected in a real environment with noise and other factors.

To make the system perform better, Wu et al. (2019) manually removed the data concerning silent recording, footstep sounds, adult speech, and other noise types. An infant's crying signals have different acoustic and prosodic information at different levels, involving vocalization, choking, coughing, and silence.

Data augmentation solves data size limitations such as noise variation, spectrogram, and signal size alternation (Ji et al., 2021). A comparison of augmentation methods was made by Fukuda et al. (2018), and for audio signal recognition, voice transformation, and noise addition, this comparison resulted in higher performance. On the other hand, feature selection is applied to eliminate redundant features that decrease the model's ability to differentiate the infant crying classification.

A fully automated segmentation algorithm implemented by Abou-Abbas et al. (2017) extracted the cry sound components in a noisy environment using the audible expiration and inspiration methods. It is studied in statistical analysis and post-processing ways based on intensity, ZCR, and feature extraction.

Yao et al. (2022) collected real-world datasets in a controlled home environment. Multiple ML approaches were proposed based on deep spectrum features (DSF) and acoustic features (AF) and trained on the built and annotated dataset. According to their studies, the model trained on the in-lab data showed low performance when tested on the data collected in a real-world environment. When tested on the RW-Filt dataset, the CNN-based model achieved the best performance, with a slight difference from their proposed model. Ji (2021) combined generated weighted prosodic and acoustic features to improve infant cry recognition. With this feature, a graph convolutional network (GCN) approach with transfer learning performed better than the CNN model in infant cry classification.

The study by Tusty et al. (2020) worked on classifying infants' cries into five classes: hunger, discomfort, stomachache, burping, and sleepiness. The model used is a combination of CNN and RNN; the former is dedicated to extracting local features from the spectrogram images, and the latter learns temporal features; at the end, there is a linear layer to perform the classification.

Infant cry audio is known for its high frequency, which ranges from 400 to 500 Hz. Liang et al. (2022) emphasized that high-frequency components with filters and stable features are obtained by cutting the signal into smaller parts. Then, the hamming window, Fast Fourier Transform (FFT), Mel filter, and, finally, the discrete cosine transform (DCT) are applied to obtain the MFCC signal. The final classification problem is solved by passing these features to the Artificial neural network (ANN), CNN, and LSTM.

Sharma et al. (2019) extracted features from infant cry audio in different forms, e.g., mean frequency, standard deviation, and median frequency. Unsupervised approaches, such as k-means clustering, hierarchical clustering, and the Gaussian mixture model (GMM), were used to perform the classification. After testing the methods with infant cry audio data, the GMM achieved the highest accuracy.

Chunyan et al. (2021) introduced a graph convolutional classification to take advantage of the potential of graph networks in unlabeled datasets and the ability to describe data points in the same class and different classes. From infant audio data, features are extracted using transfer learning with ResNet50 as a base model, and the semi-supervised and supervised graphs are constructed with labeled training data and unlabeled testing data, respectively. The researchers achieved a better result than the CNN model on both the semi-supervised and supervised GCN.

Multi-class classification on infant cry data was performed by Vincent et al. (2021) with pain, hunger, and sleepiness classes. The audio signal was primarily converted to a spectrum image using an STFT method. Automatic feature extraction was performed by providing the image to the CNN. The extracted features were input to the SVM classifier to obtain the final class. Different SVM kernels were evaluated. The highest accuracy, 88.89%, was obtained from the RBF-SVM.

Researchers conducted a study on classifying infant crying into four categories: hunger, pain, tiredness, and diaper (Joshi et al., 2022). They first preprocessed the signals and converted them into Mel-spectrograms.

After that, they explored the possibility of using a CNN-based DL approach with different architectures, like VGG16 and Yolov4. The researchers also proposed a multi-stage heterogeneous stacking ensemble model using four classifiers, each corresponding to a different level. The researchers also proposed a multi-stage heterogeneous stacking ensemble model using four classifiers, each corresponding to a different level. Their motivation is that the dataset is diverse and contains spectrograms of various audio frames, hence a single classifier cannot perform well. For the different levels, they used different model types like SVM, MLP, NuSVC, RF, XGBoost, and AdaBoost, but ultimately settled on NuSVC, RF, AdaBoost, and XGBoost for the four levels in order. The researchers found their proposed approach achieved an average accuracy of 92% and an average F1 score of 0.923, which were significantly higher than the CNN-based DL approach, which had an accuracy of 75%.

Multimodal data were collected to investigate the correlation between the newborn distress levels and neurological diseases (Laguna et al., 2023). Various data sources, such as voice recordings, electroencephalogram (EEG), near-infrared spectroscopy (NIRS), facial expressions, and body movements, were utilized. The data was recorded under three conditions: resting, crying, and distress. Different filters and preprocessing techniques were applied based on the data source. Experts validated all data. Finally, a total of 1,473 crying and 491 distressed crying samples were collected. Both the ML and DL algorithms were used to classify distress vs. non-distress. A CNN-based model achieved a higher accuracy of 93%, outperforming the RF-based model, which had an accuracy of 89%. Additionally, the importance of each feature was evaluated using various algorithms, including ANOVA, Tukey–Kramer, Mann–Whitney *U*-test, and Kruskal–Wallis. A correlation was found between most of the extracted features from the signals, including fundamental frequency, brain activity (delta, theta, and alpha frequency bands), cerebral and body oxygenation, heart rate, facial tension, and body rigidity. The study concluded that infant cries could be a biomarker for detecting specific pathologies.

To classify infant cry audio signals into those that were normal and abnormal, Hariharan et al. (2012) used time-frequency derived features with short-time Fourier-transform (STFT) and general regression neural network (GRNN) methods for the classification task, which performed better than multi-layer perceptron (MLP) and the time-delay neural network (TDNN).

## 3 Materials and methods

**Figure 1:**
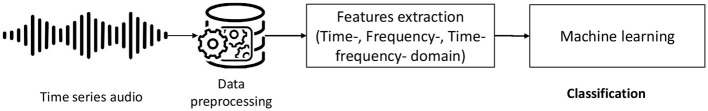
Methodology block diagram.

Our proposed methodology is illustrated in Figure 1. The raw audio is processed in a predefined form, such as a 5-s duration and frequency sampling rate of 22,050 Hz. The features are extracted from the resulting signal. Different features in time (ZCR and RMS), frequency, and time-frequency domain (MFCC) are extracted. Moreover, MFCC-based TSI is used to transform MFCC into images.

### 3.1 Dataset

Multiple datasets exist for infant crying, such as the Chillanto dataset (Reyes-Galaviz et al., 2008) and donate-a-cry-corpus.^1^ Infants in both datasets have five statuses. There is also another type (Yao et al., 2022) that is collected in real life and includes noise and adult speech.

In our work, a “donate a cry-corpus” dataset was used for model training and validation as it was publicly available. The dataset consists of 457 signals with five classes: hungry, burping, tired, belly pain, and discomfort, as listed in Table 1.

**Table 1:** Dataset distribution between the classes.

**Donate a cry-corpus**	**Hungry**	**Tired**	**Burping**	**Belly pain**	**Discomfort**	**Total**
Original	382	24	8	16	27	457
Augmented data	382	384	384	384	405	1,939

### 3.2 Data preparation

The dataset used in this study is small and characterized by a high imbalance between the classes; hence, we took certain measurements to avoid misclassification and minimize the variance-bias trade-offs that may occur due to this. Several techniques can be used in this case, such as undersampling the class with higher data and oversampling the class with the lowest data points. In the current ML trend, mainly in DL, data augmentation has proven to be more effective in image and audio data and is widely used. Data augmentation in audio data has the importance of improving the model's performance by increasing the size of the data and introducing different variants of a single data entity. Gaussian noise was applied in our experiment with a minimum and maximum amplitude of 0.01 and 0.015, respectively. As the hungry class already has a larger sample size, the augmentation is applied to the rest of the classes independently for each technique. The outcome from data augmentation is listed in Table 1.

### 3.3 Feature extraction

Audio features can be extracted in the time-, frequency-, or time-frequency domains. Time-domain features are extracted from the raw audio. By contrast, the frequency-domain features are extracted after converting the raw audio from the time domain into the frequency domain using Fourier transform. In the time-frequency domain, Fourier transform is applied to the time-domain waveform. ZCR, amplitude envelope, and RMS energy are examples of time domain features. Contrarily, spectrogram/Mel- spectrogram, band energy ratio, spectral flux, and spectral centroid are examples of frequency-domain features. MFCCs and constant Q transforms are examples of time-frequency-domain features. In our study, ZCR, RMS, Mel-spectrogram, and MFCCs were used as features to classify the status of the infants. Moreover, MFCC features were transformed into images using TSI. The following sections introduce a brief description of these features.

#### 3.3.1 Time-domain features

In audio, ZCR refers to the number of times the audio waveform crosses the zero axis per second. It measures the frequency of changes in the polarity of the audio signal. A higher ZCR indicates a more rapidly changing audio waveform, whereas a lower ZCR indicates a more slowly changing waveform. In audio, RMS refers to the measure of the average power of an audio signal. It is calculated by taking the square root of the mean (average) of the squared values of the audio signal. RMS is a more accurate measure of the loudness or amplitude of an audio signal than peak amplitude, as it considers the entire waveform rather than just the highest point.

#### 3.3.2 Time-frequency domain features

Before going further, a cepstrum represents the information of the changing rate in spectral bands. Mathematically, it is the spectrum of the log of the spectrum of the time signal. The obtained spectrum is not in frequency or time but in the quefrency domain. Therefore, the MFCC represents the coefficients of the Mel-frequency spectrum. The MFCC is used to model features of the audio signal (Abdul and Al-Talabani, 2022). It extracts harmonics and side bands of the signal's spectrum. The MFCC includes several steps: pre-emphasis, framing and windowing, DFT, Mel-frequency filter bank, logarithm, and DCT. The parameters used for a feature generation with the MFCC are as follows: number of features = 20; number of Mel bands = 20; FFT window = 1,024; and a band-pass filter of (300, 600) Hz.

#### 3.3.3 Frequency domain features

**Figure 2:**
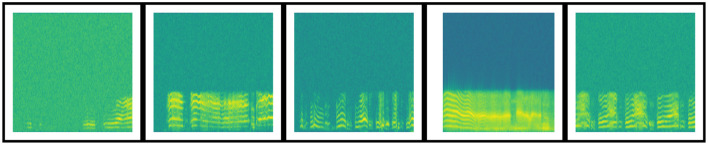
Visualization of Mel-spectrograms of different infant crying statuses.

A spectrogram visually represents a time-series signal or signal strength. Spectrograms can be linear or Mel-spectrograms. The linear spectrogram is efficient when all frequency components are equally important. On the contrary, the Mel-spectrogram models non-linear perception, such as human hearing. A 216 × 216 Mel-spectrogram was generated as features. The Mel-spectrogram of a few samples from different infant classes is presented in Figure 2. The following settings were used to generate the Mel-spectrogram in Python's Matplotlib library: signal length = 5 s; sampling rate = 22,050 Hz; window, Hanning window with overlapping of 128; 256 data points for FFT generation.

#### 3.3.4 Time-series imaging algorithms

**Figure 3:**
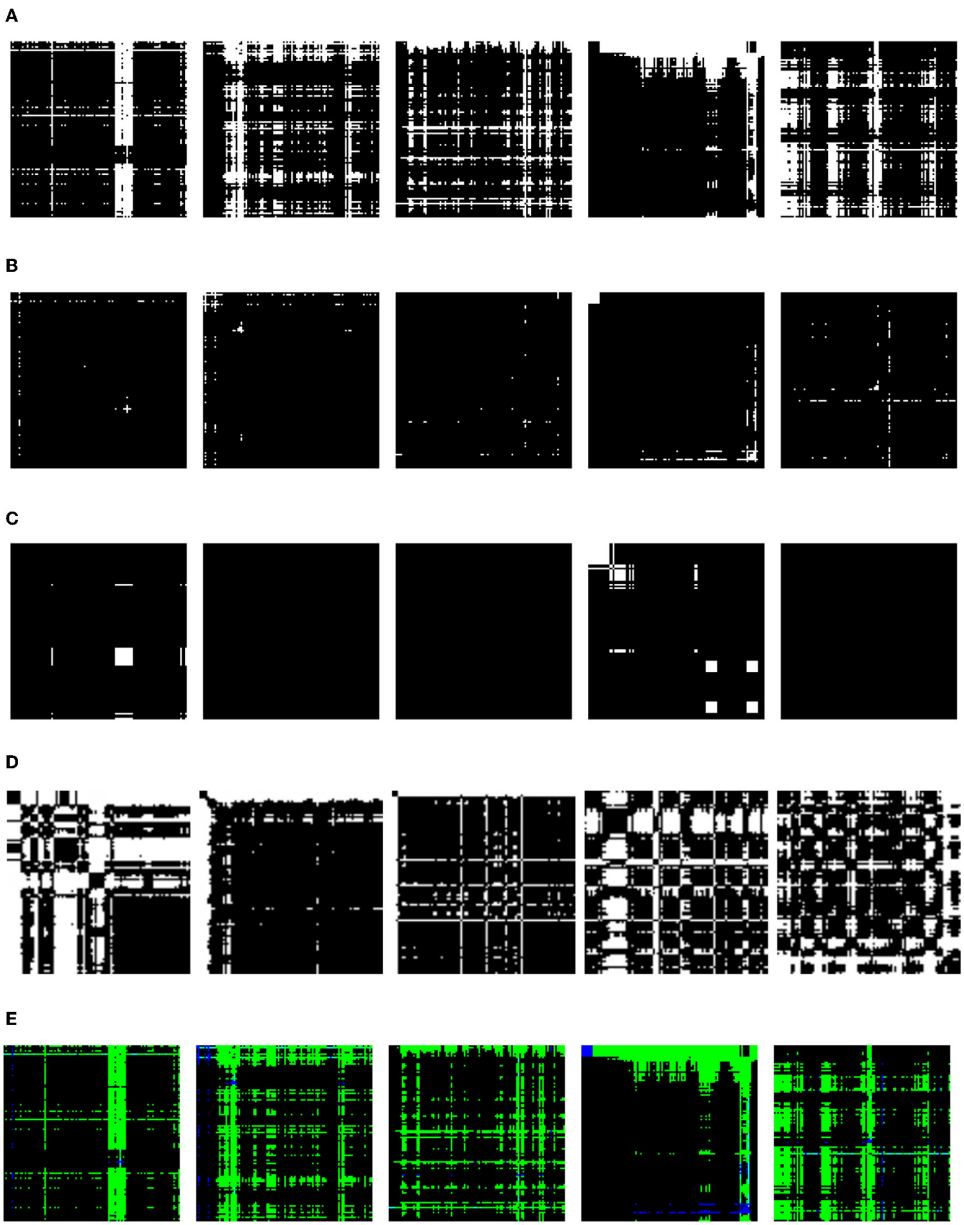
Visualization of different TSI algorithms. Columns represent the infant crying statuses: belly pain, burping, discomfort, hungry, and tired. **(A)** GADF. **(B)** GASF. **(C)** MTF. **(D)** RP. **(E)** RGB GAF.

TSI algorithms transform time-series signals into images (Wang and Oates, 2015), as shown in Figure 3. They analyze time-series data and can provide valuable insights into trends and patterns that may not be visible through other analysis methods. The obtained images from TSI can be treated as typical image recognition tasks. These techniques can also benefit from CNN's approach to solving image classification problems. TSI can be obtained using different algorithms, such as Gramian angular summation fields (GASF), Gramian angular difference fields (GADFs), recurrence plots (RP), Markov transition fields (MTF), and RGB GAF. GADFs calculate the temporal correlation between the pairs of time-series values. RGB GAF is based on images obtained from GASF and GADF. MTF finds a field of transition probabilities for time-series data. RP calculates pairwise Euclidean distances between time-series data. The authors investigated the performance of RP with CNN (Hatami et al., 2018). This approach outperformed the existing deep architectures and SOTA in time-series classification (TSC).

### 3.4 Implementation details

The features extracted, such as the ZCR, RMS, Mel-spectrogram, and MFCCs, and, additionally, image data obtained from MFCCs using time-series imaging algorithms, were supplied to ML algorithms.

**Figure 4:**
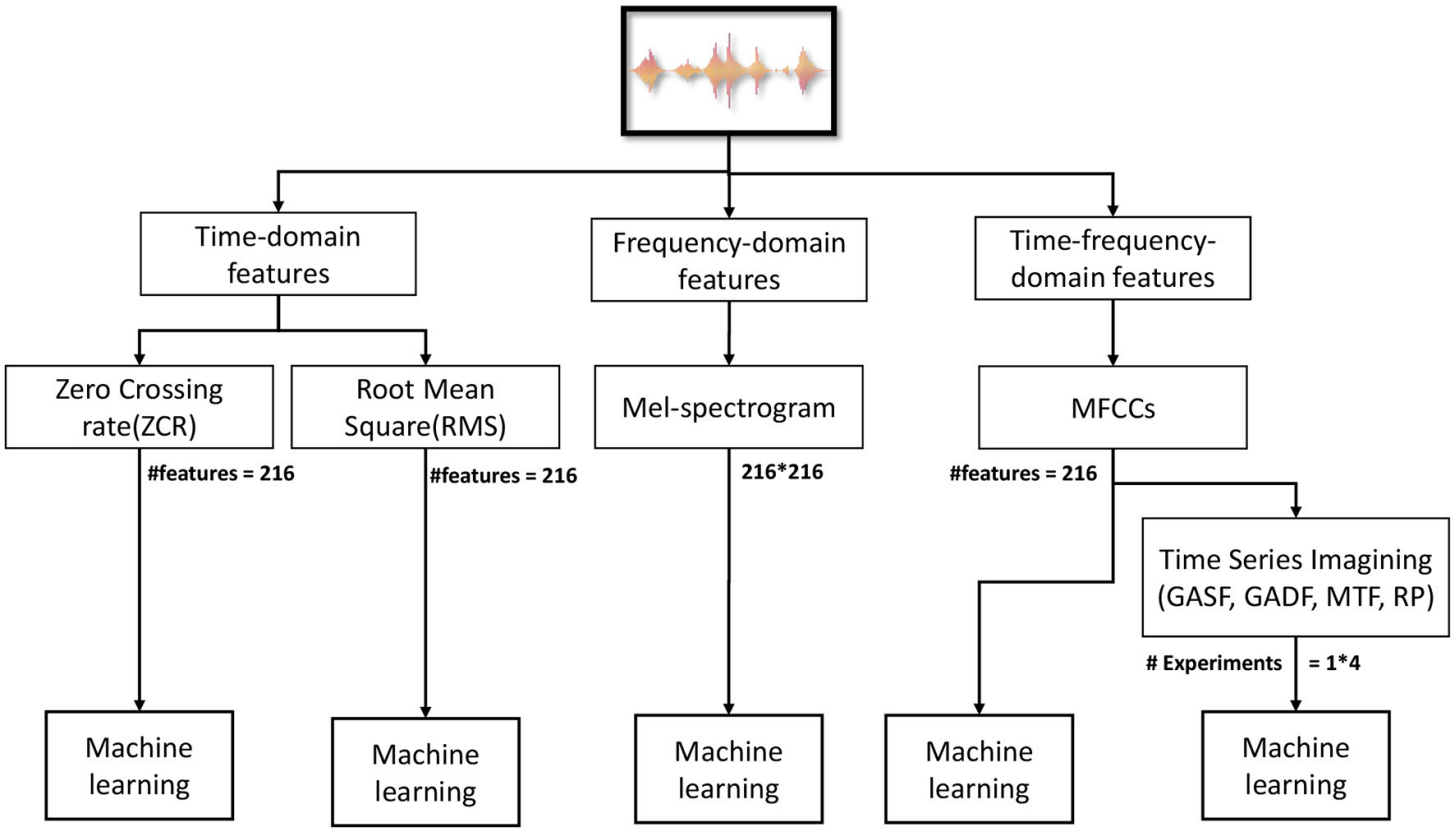
Different features for the model in the experiments conducted.

All conducted experiments are illustrated in Figure 4. We used several algorithms, such as LR, Ridge, SVM, DT, RF, K nearest neighbors (KNN), eXtreme Gradient Boosting (XGB), and Naive Bayes (NB). We used Python for programming^2^ and API Librosa 0.9.2^3^ to process the audio data and OpenCV for image processing. For time-series imaging implementation, PyTs library^4^ was used.

The data is split into 80% training and 20% testing. Categorical cross-entropy was used as a loss function. The hyperparameter tuning technique was used with grid search-based cross-validation to find the best model for validation accuracy. A K fold of *k* = 10 was used to validate the models.

The audio duration was 5 s, and 216 features were used from ZCR, RMS, and MFCCs. Regarding image-based models, we used 216 × 216 images for Mel-spectrogram and time-series imaging techniques.

We used signals with a fixed duration of 5 s. The frame length was set to 2048 and the hop length to 512 in the Librosa library implementation, producing 213 frames, which can be calculated using Equation 1. Additionally, the library added some padding; therefore, we finally ontained 216 frames for each signal.


(1) $$\begin{array}{l} Frames\;number=\;\frac{Signal\;length\;-\;\left(Frame\;length\;-\;hop\;length\right)}{Frame\;length\;-\;\left(Frame\;length\;-\;hop\;length\right)} \end{array}$$

It is worth mentioning that while generating MFCC features for the first 5 s from the audio signal, we used a band-pass filter of (300, 600) Hz on the infant signals. Additionally, we used 20 Mel bands and the length of the FFT window was 1,024, while the other parameters were taken by default from the Librosa library.

In this study, we used hyperparameter tuning using grid search while training ML models. For convenience, the parameter space is not added to the paper. All settings are provided in the source code. In grid search, we trained models using all possible combinations of parameters. The best model (for each experiment) was selected and reported.

Model results on test data were reported in terms of accuracy, macro f1-score (f1-score), macro precision (PRC), and macro recall (REC), as listed in Equations (2–5).


(2) $$\begin{array}{l} Accuracy\;\left(ACC\right)=\frac{TP\;+\;TN}{TP\;+\;TN\;+\;FP\;+\;FN} \end{array}$$

(3) $$Macro\;f1score\;=\frac{\sum_{i=1}^{n}F\;1\;score_{i}}{n}$$

(4) $$\begin{array}{l} f1score=\;2\;\times \frac{PRC\;\times \;REC}{PRC\;+\;REC} \end{array}$$

(5) $$\begin{array}{l} PRC\;=\frac{TP}{TP\;+\;FP} \end{array}$$

(6) $$\begin{array}{l} REC\;=\frac{TP}{TP\;+\;FN} \end{array}$$

TP, TN, FP, and FN are true positive, true negative, false positive, and false negative, respectively.

## 4 Results and discussion

The solution proposed was evaluated on the infant cry data discussed in the dataset subsection. The testing result was obtained from features extracted by MFCCs, the Mel-spectrogram, ZCR, RMS methods, and several ML classification algorithms, such as XGB, LR, and NB, as listed in Table 2. Next, the test result obtained from different classifiers with features extracted by several TSI methods is shown in Table 3. Some previous studies tested their solution on the dataset we used, and in Table 4, a performance comparison was made with our result. The highest accuracy obtained with the MFCC feature is in the random forest classifier and support vector machine, at ~96%. Feature extraction by ZCR seems to have the same performance with the RF and KNN methods but a slightly lower performance than MFCC features. Regarding the RMS feature extraction method, the SVM classifier achieved the best accuracy. We have also explored TSI methods for feature extraction, as shown in Table 3, and tested them in four ML classifiers. The GADF method and bagging classifier resulted in the highest accuracy in this part of the experiment. Mel-spectrogram feature representation also resulted in high precision when tested in a bagging classifier.

**Table 2:** Evaluation (testing) results: MFCC-, ZCR-, and RMS-based ML.

	**PRC (%)**	**REC (%)**	**ACC (%)**	**F1-score (%)**
**MFCC**				
LR	91.24	90.00	90.21	88.92
Ridge	77.06	78.28	78.35	77.24
DT	93.83	93.56	93.56	93.61
RF	96.79	96.41	**96.39** ^*^	96.46
NB	55.42	45.71	45.62	44.24
KNN	94.33	94.33	94.33	94.33
XGB	95.10	95.10	95.10	95.10
SVM	96.13	96.13	**96.13**	96.13
Bagging	94.85	94.85	94.85	94.85
**ZCR**				
LR	92.64	92.47	92.53	92.53
Ridge	95.65	95.12	95.10	95.21
DT	90.16	89.94	89.95	90.01
RF	96.52	95.90	**95.88**	95.98
NB	66.42	67.31	67.01	65.61
KNN	96.52	95.90	**95.88**	95.98
XGB	95.26	94.85	94.85	94.92
SVM	96.21	95.63	**95.62**	95.72
Bagging	96.18	95.64	**95.62**	95.70
**RMS**				
LR	87.72	87.34	87.37	87.43
Ridge	86.63	86.33	86.34	86.35
DT	73.61	73.08	72.94	73.06
RF	91.27	91.19	91.24	91.21
NB	65.86	64.63	64.43	64.43
KNN	90.81	90.88	90.98	90.76
XGB	90.21	88.87	88.92	89.06
SVM	95.62	95.35	**95.36**	95.40
Bagging	92.00	92.00	92.01	91.99

Bold values indicate the best performance obtained by the authors in their experiments. Bold values with ^*^ indicate the highest performance obtained among all studies.

**Table 3:** Evaluation (testing) results: MFCC-TSI-based.

	**PRC (%)**	**REC (%)**	**ACC (%)**	**f1-score (%)**
**GADF**				
DT	69.77	70.28	70.36	69.25
RF	91.42	91.12	91.24	90.76
KNN	93.30	93.19	93.30	93.07
Bagging	92.98	92.97	93.04	92.89
**GASF**				
DT	59.57	47.16	46.91	45.76
RF	78.16	77.20	77.32	76.79
KNN	70.36	70.36	70.36	70.36
Bagging	83.52	83.69	83.76	83.32
**MTF**				
DT	71.33	53.56	53.35	55.19
RF	77.37	62.03	61.86	63.69
KNN	85.78	58.52	58.25	60.28
Bagging	77.90	63.07	62.89	64.74
**RP**				
DT	66.07	66.28	66.24	66.00
RF	89.06	89.08	89.18	88.87
KNN	91.22	90.89	90.98	90.72
Bagging	92.25	92.22	92.27	92.22
**RGB GAF**				
DT	62.83	61.33	61.34	61.49
RF	86.77	86.69	86.86	86.18
KNN	92.30	92.16	92.27	92.02
Bagging	91.25	91.13	91.24	90.94
**Mel-spectrogram**				
DT	80.88	80.82	80.67	80.49
RF	**94.92**	94.36	94.33	94.43
KNN	**94.55**	94.09	94.07	94.14
Bagging	**95.45** ^*^	94.85	94.85	94.95

Bold values indicate the best performance obtained by the authors in their experiments. Bold values with ^*^ indicate the highest performance obtained among all studies.

**Table 4:** Comparative study of the performance of this study with state-of-the-art research.

**Features**	**Model**	**Donate a cry corpus**			
		**PRC (%)**	**REC (%)**	**ACC (%)**	**F1-score (%)**
Hand-crafted features (F1, F2, F3, MFCC, and LPCC; Ozseven, 2023)	SVM	87.88	87.58	**87.87**	87.72
	RNN	72.09	75.19	74.84	73.01
	PNN	88.95	85.69	87.17	87.14
Spectrogram (Ozseven, 2023)	GoogleNet	51.29	43.42	53.52	44.38
	ShuffleNet	93.12	98.05	92.25	91.91
	ResNet-18	93.46	93.40	**93.71**	93.43
Scalogram (Ozseven, 2023)	GoogleNet	64.35	58.62	65.63	64.05
	ShuffleNet	95.18	93.78	94.56	94.45
	ResNet-18	94.71	94.81	**95.17**	94.75
Frequency, entropy, spectral + GMM (Sharma et al., 2019)	–	–	–	**81.27**	–
Our work					
MFCC + GADF	KNN	93.30	93.19	93.29	93.07
MFCC + GASF	KNN	73.06	73.06	73.06	73.06
MFCC + MTF	Bagging	77.89	63.07	62.89	64.74
MFCC + RP	Bagging	92.25	92.22	92.27	92.22
MFCC + RGB GAF	KNN	92.30	92.16	92.27	92.02
Mel-spectrogram	Bagging	95.45	94.85	94.85	94.95
MFCC	RF	96.79	96.41	**96.39** ^*^	96.46
MFCC	SVM	96.13	96.14	**96.13**	96.13
ZCR	RF	96.52	95.89	**95.88**	95.98
ZCR	KNN	96.52	95.89	**95.88**	95.98
ZCR	SVM	96.21	95.63	**95.62**	95.72
ZCR	Bagging	96.18	95.64	**95.62**	95.70
RMS	SVM	91.99	91.99	**95.36**	95.40

Bold values indicate the best performance obtained by the authors in their experiments. Bold values with * indicate the highest performance obtained among all studies.

The final goal of this study was to bring a solution to the research community with better performance than previous studies. Ozseven (2023) conducted state-of-the-art research and studied the extraction of infant cry signals. The author tested their solution on different ML and DL pretrained models. They obtained the highest accuracy with hand-crafted features and the SVM classifier. The accuracy of the pretrained ResNet-18 model train with spectrogram-represented features was better than the former. Again, they trained the ResNet-18 model with the scalogram feature, and finally, they achieved their best accuracy of 95.17%. Our study has shown performance improvement over previous state-of-the-art studies compared with different metrics.

After conducting several experiments, we have selected and presented the best results in Table 4. As can be observed in the table, MFCC features trained on random forest and support vector machine classifiers, and ZCR features trained on RF, KNN, SVM, and bagging classifiers have all yielded higher precision, recall, accuracy, and f1-score results than SOTA on a donate-a-cry corpus dataset. In addition, RMS features trained on SVM were more accurate and had a higher f1-score than previous results. One of the time-series imaging algorithms, GADF, achieved an accuracy of more than 93%, which shows that it has the power to represent this type of data in image format and yield good performance. If the signal duration was sufficiently long, GADF could find a better temporal correlation and, consequently, achieve higher accuracy. To sum up, the highest accuracy, precision, recall, and f1-score are obtained from the MFCC feature with an RF classifier.

## 5 Conclusion

Infant cry classification is essential for helping inexperienced parents understand the feelings of their child. It can be used for the medical diagnosis of infant developmental health. The crying of an infant is their only way of communicating with the world. Understanding their crying helps caregivers understand their needs. This research proposes an ML model based on different features. The proposed method successfully classifies five classes of infant crying, such as hunger, discomfort, belly pain, burping, and tiredness. The best performance is achieved with the MFCC-based ML model, with an accuracy of 96.39% on testing data, outperforming SOTA. Even though the models obtained great classification accuracy with a signal duration of 5 s of infant crying, their performance might suffer when evaluated on longer signal durations. As a result, we intend to investigate the feasibility of combining characteristics such as ZCR, RMS, and MFCCs in a single model to attain high accuracy irrespective of signal duration. Other datasets, more advanced data augmentation techniques, and alternative DL-based methodologies will also be considered.

## Data availability statement

Publicly available datasets were analyzed in this study. This data can be found at: https://github.com/gveres/donateacry-corpus. The code is available at https://github.com/Hammoudmsh/ML_based_infant_Crying_classification.git.

## Author contributions

MH: Conceptualization, Data curation, Investigation, Writing – original draft. MG: Formal analysis, Software, Validation, Writing – original draft. AB: Funding acquisition, Validation, Visualization, Writing – review & editing. AS: Methodology, Project administration, Resources, Supervision, Writing – review & editing.

## Footnotes

^1^ https://github.com/gveres/donateacry-corpus

^2^ https://www.python.org/

^3^ https://librosa.org/

^4^ https://pyts.readthedocs.io/en/stable/modules/image.html
